# MiR-3470b promotes bovine ephemeral fever virus replication via directly targeting mitochondrial antiviral signaling protein (MAVS) in baby hamster Syrian kidney cells

**DOI:** 10.1186/s12866-018-1366-6

**Published:** 2018-12-27

**Authors:** Peili Hou, Hongmei Wang, Guimin Zhao, Guixue Hu, Xianzhu Xia, Hongbin He

**Affiliations:** 1 College of Animal Science and Technology, Jilin Agricultural University, Changchun, 130118 People’s Republic of China; 2 Key Laboratory of Animal Resistant Biology of Shandong, Ruminant Diseases Research Center, College of Life Sciences, Shandong Normal University, Jinan, 250014 People’s Republic of China; 3 Key Laboratory of Jilin Province for Zoonosis Prevention and Control, Institute of Military Veterinary, Academy of Military Medical Sciences, Changchun, 130122 People’s Republic of China

**Keywords:** Bovine ephemeral fever virus (BEFV), MiR-3470b, Mitochondrial antiviral signaling protein (MAVS), Virus replication

## Abstract

**Background:**

Bovine ephemeral fever virus (BEFV), the causative agent of bovine ephemeral fever, is an economically important pathogen of cattle and water buffalo. MicroRNAs (miRNAs) are endogenous 21-23 nt small non-coding RNA molecules that binding to a multiple of target mRNAs and functioning in the regulation of viral replication including the miRNA-mediated antiviral defense. However, the reciprocal interaction between bovine ephemeral fever virus replication and host miRNAs still remain poorly understood. The aim of our study herein was to investigate the exact function of miR-3470b and its molecular mechanisms during BEFV infection.

**Results:**

In this study, we found a set of microRNAs induced by BEFV infection using small RNA deep sequencing, and further identified BEFV infection could significantly up-regulate the miR-3470b expression in Baby Hamster Syrian Kidney cells (BHK-21) after 24 h and 48 h post-infection (pi) compared to normal BHK-21 cells without BEFV infection. Additionally, the target association between miR-3470b and mitochondrial antiviral signaling protein (MAVS) was predicted by target gene prediction tools and further validated using a dual-luciferase reporter assay, and the expression of MAVS mRNA and protein levels was negatively associated with miR-3470b levels. Furthermore, the miR-3470b mimic transfection significantly contributed to increase the BEFV N mRNA, G protein level and viral titer, respectively, whereas the miR-3470b inhibitor had the opposite effect on BEFV replication. Moreover, the overexpression of MAVS or silencing of miR-3470b by its inhibitors suppressed BEFV replication, and knockdown of MAVS by small interfering RNA also promoted the replication of BEFV.

**Conclusions:**

Our findings is the first to reveal that miR-3470b as a novel host factor regulates BEFV replication via directly targeting the MAVS gene in BHK-21 cells and may provide a potential strategy for developing effective antiviral therapy.

**Electronic supplementary material:**

The online version of this article (10.1186/s12866-018-1366-6) contains supplementary material, which is available to authorized users.

## Background

Bovine ephemeral fever (BEF), known as “three day sickness”, is an arthropod-borne and acute viral infection of cattle and water buffalo. It can spread rapidly and lead to considerable economic losses due to decreased-milk production, reduced-male fertility, disruption of stock movement, and even causes death of infected animals [1, 2]. In addition, BEF is believed to be an ancient disease that has been endemic in tropical, subtropical, and temperate regions of Africa, Asia, and Australia [3–5]. Its causative agent, bovine ephemeral fever virus (BEFV) is an enveloped, nonsegmented, single stranded negative sense RNA virus in the *Ephemerovirus* genus of the *Rhabdoviridae* family. Nevertheless, in terms of pathology, the underlying pathogenic mechanisms of BEFV have not largely been elucidated.

MicroRNAs (miRNAs) are a class of endogenous and short noncoding RNAs (approximately 22 nucleotides in length) processed from endogenous transcripts. Generally, microRNAs have emerged as master regulators that typically bind to complementary sequences in the 3′ untranslated region (UTR) of multiple mRNA targets and modulate gene expression either via the translational inhibition or degradation of their target messenger RNAs (mRNAs) in a sequence-specific manner [6–8]. Furthermore, miRNA interacts with mRNA mainly through the seed region defined as nucleotides 2–8 from 5′ end of the sequence of miRNA [8]. Meanwhile, a variety of published data showing that miRNAs encoded by DNA viruses are conducive for developing a beneficial environment to viral replication [9, 10]. Indeed, there is now overwhelming evidence that host miRNAs are not only implicated in the maintenance of normal cellular processes [11, 12], and they also play important regulatory and ubiquitous roles in signaling pathways involved in various viral infection, replication, and pathogenicity by regulating genes expression [11, 13, 14]. Recently, it is the hotspot to study the relationship between miRNA regulation and virus infection.

Mitochondrial antiviral signaling protein (MAVS), also known as virus-induced signaling adaptor (VISA), IFN-β promoter stimulator (IPS-1) and CARD adaptor inducing IFN-β (Cardif), is a key adaptor protein that plays a central role in mediating retinoic acid-inducible gene I (RIG-I) and MDA5-dependent antiviral responses signaling, which has been extensively studied in mammals for its important role of activation of latent transcription factors and IFN-α/β production. Furthermore, MAVS appears to be implicated in other signaling cascades such as apoptosis and other pro-inflammatory cytokines [15–17]. As MAVS works as a vital adaptor of mediating effective responses against a variety of DNA or RNA viruses, multiple strategies have been used to restrict host innate immune responses by regulation or modification of MAVS to facilitate its infection, including phosphorylation, ubiquitination [18, 19]. Beyond conventional protein modification, currently, numerous research studies have indicated that a decisive role for microRNAs in modulating viral pathogenesis either by directly altering MAVS gene expression and subsequently antiviral immune response. For instance, miR-22, miR-125a (or -b) and miR3570b have been indicated to participate in modulating the expression of MAVS upon Japanese encephalitis virus (JEV), influenza A virus (IAV) and rhabdovirus in teleost fish infections, respectively [20–22].

In view of the above-mentioned facts that many molecules and miRNAs are involved in modulating viral replication, however, the mechanism by which BEFV inhibits MAVS expression is not yet established. In the present study, we investigated the role of miR-3470b in regulation of MAVS and examined its consequences on BEFV infection. We found that miR-3470b was up-regulated during BEFV infection through high-throughput sequencing and it was further validated by RT-qPCR. Then, BHK-21 cells were transfected with the miR-3470b mimics or inhibitor to evaluate whether miR-3470b expression had potential effects on BEFV replication. Subsequently, miR-3470b targeting its response elements (MREs) in the 3’UTR of MAVS was predicted and further validated using a dual-luciferase reporter assay. And the negative expression levels between MAVS and miR-3470b were verified. Furthermore, effect of MAVS gene expression on BEFV replication was determined. Our findings underline one of the potential mechanisms of miR3470 in controlling BEFV replication and introduce MAVS as a key mediator of this response.

## Results

### BEFV infection up-regulates miR-3470b expression

**Fig. 1:**
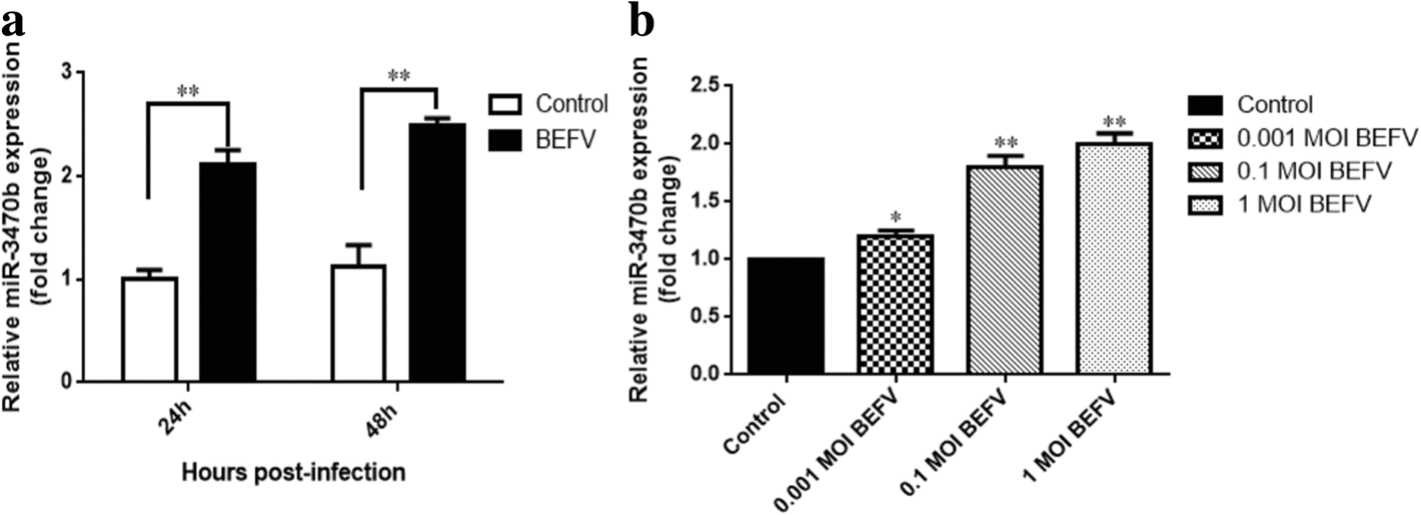
BEFV infection up-regulate miR-3470b expression. (**a**) Relative quantitation RT-PCR analysis of endogenous miR-3470b in BHK-21 cells infected with BEFV (MOI of 0.1) for 24 h and 48 h. (**b**) Relative quantitation RT-PCR analysis of endogenous miR-3470b in BHK-21 cells infected with different doses of BEFV (MOI of 0.01, 0.1, 1). Error bars denote the SD from at least three independent experiments. * *P* < 0.05; ** *P* < 0.01

In order to study the effect of BEFV infection on host response, we conducted a set of microRNAs expression profile analysis of BEFV infected BHK-21 cells for 24 h using high-throughput technologies. Differential miRNA expression analysis (DR ≤ 0.05 and | log_2_FC | ≥ 1) showed that 524 miRNAs were up-regulated and 41 miRNAs were down-regulated (Additional file 1). Among those differentially expressed miRNAs, we firstly focused on the up-regulated miRNAs that have potential to inhibit gene expression in BEFV-infected cells compared with control, and miR-3470b was one of the top-ranked hits. To further investigate the differentiated expression of miR-3470b during the BEFV infection, we performed miRNA quantification in the BHK-21 cells after infection for 24 h and 48 h by RT-qPCR. The results revealed that the relative expression level of miR-3470b was significantly higher after BEFV infection compared with control (Fig. 1a). Furthermore, the relationship between miR3470b expression and BEFV infection was a viral dose-dependent increase effect (Fig. 1b). Thus, these results demonstrated that miR-3470b might play a role in the process of BEFV infection.

### MiR-3470b promotes BEFV replication in BHK-21 cells

**Fig. 2:**
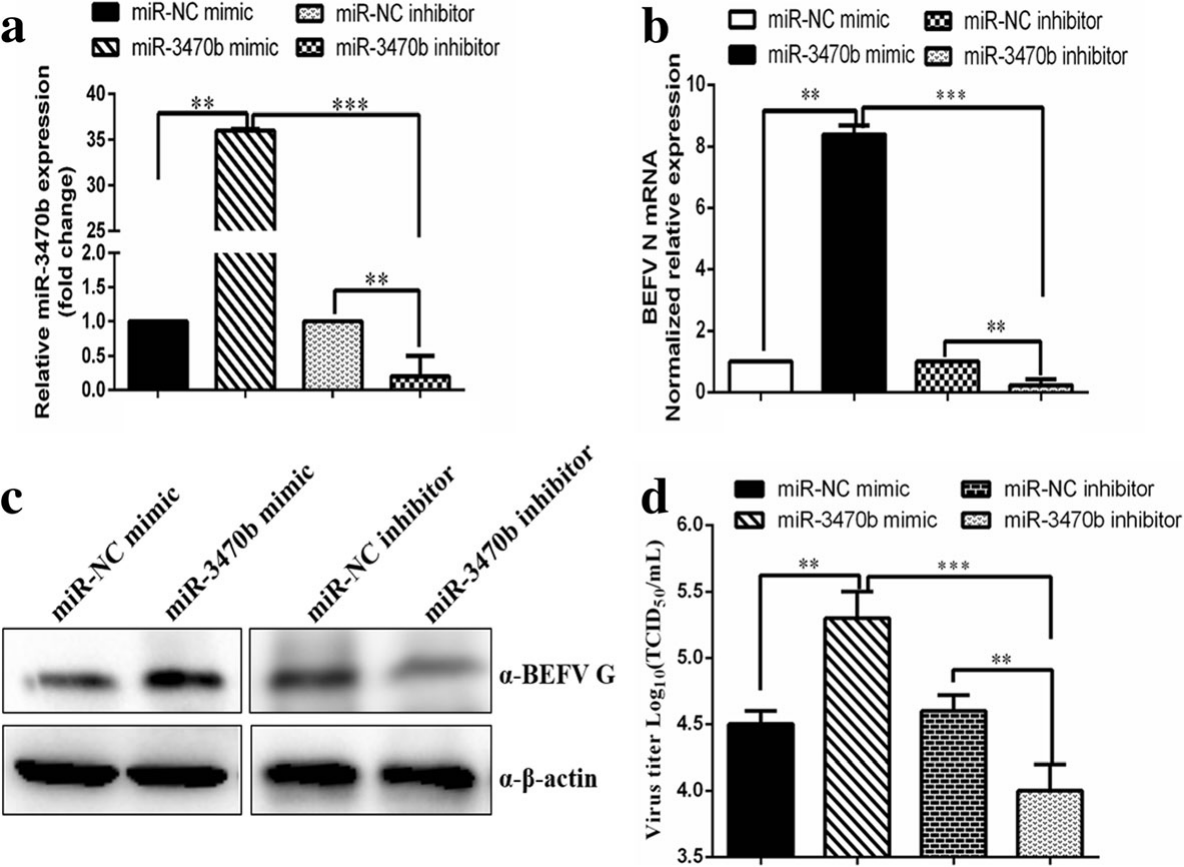
MiR-3470b promotes BEFV replication in BHK-21 cells. **a** BHK-21 cells were transfected with 50 nM miR-3470b mimic, 50 nM miR-3470b inhibitor and 50 nM irrelevant-targeting negative control groups (miR-NC mimic and miR-NC inhibitor) for 36 h respectively. The efficiency for transfection of the miR-3470b mimics and miR-3470b inhibitor were detected by SYBR Green based RT-qPCR assay. **b** BEFV genome N mRNA levels were determined by relative quantitative RT-PCR assay and fold changes were calculated using the 2^-ΔΔCt^ method. All data were representative of three independent experiments and presented as means ± SD. ***p < 0.001, **p < 0.01, and *p < 0.05. **c** BEFV G protein levels were detected by western blot with anti-G polyclone antibody. Numbers below the image was used for quantifications of protein blot intensities by gray value analysis derived from image J software. **d** BHK-21 cells were inoculated with 0.1MOI BEFV after transfection with 50 nM of miR-3470b mimic, miR-NC mimic, miR-3470b inhibitor and miR-NC inhibitor respectively for 24 h. BEFV titers were determined by TCID_50_ with the Reed-Muench endpoint method

To evaluate whether miR-3470b has potential effects on BEFV replication, the BHK-21 cells were transfected with the miR-3470b mimics, NC mimics, miR-3470b inhibitor or NC inhibitor. First, we analyzed the efficacy of the miR-3470b mimics or miR-3470b inhibitor in regulating the expression level of miR-3470b. Compared to the cells transfected with the NC groups, a significant increase of the miR-3470b level transfected with the miR-3470b mimics, and an obvious inhibition of miR-3470b inhibitor on the expression of miR-3470b were observed in the BHK-21 cells (Fig. 2a). Then the BHK-21 cells pre-transfected with the miR-3470b mimics, inhibitor or corresponding NC groups were infected with BEFV for 24 h. The cells were harvested post-infection and subjected to real-time RT-PCR, western blotting and virus titration analysis of BEFV. As shown in Fig. 2b and c both mRNA levels of BEFV N gene and BEFV G protein levels were significantly increased in miR-3470b mimic transfected cells compared to transfected BHK-21 cells with NC mimic. Moreover, the viral titer (shown as the lgTCID_50_/ml) was significantly enhanced within in miR-3470b mimic transfected BHK-21 cells compared to NC mimic control groups (Fig. 2d). By contrast, knockdown of miR-3470b by inhibitors reduced BEFV replication (Fig. 2b-2d). Collectively, these results indicated that the miR-3470b expression could contribute to BEFV production.

### MiR-3470b down-regulates MAVS expression by directly targeting its 3’UTR

**Fig. 3:**
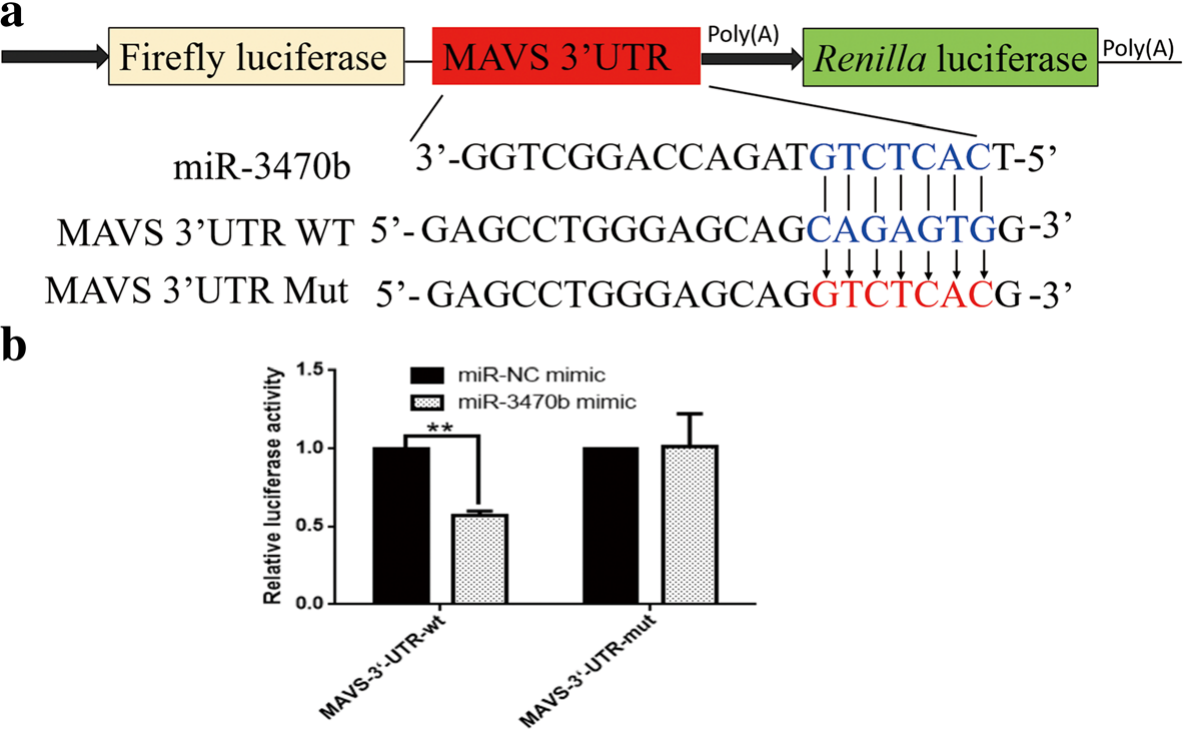
MAVS is one of the targets of miR-3470b. **a** Schematic of the reporter gene construction and the putative seed matches between miR-3470b and the MAVS 3’UTR. The MAVS 3’UTR contain wild or mutant types of the seed match of miR-3470b constructed and inserted into the luciferase reporter pmirGLO vector are indicated. **b** 50 nM of the indicated miRNA mimics or 50 nM of the indicated miRNA NC mimic was co-transfected with 50 ng of the indicated reporter constructs in 293 T cells. Firefly and *Renilla* luciferase activity levels were measured at 24 h post-transfection. The activity of firefly luciferase was normalized to that of *Renilla* luciferase. The data are representative of three independent experiments and presented as means ± SD. **p < 0.01, and *p < 0.05

**Fig. 4:**
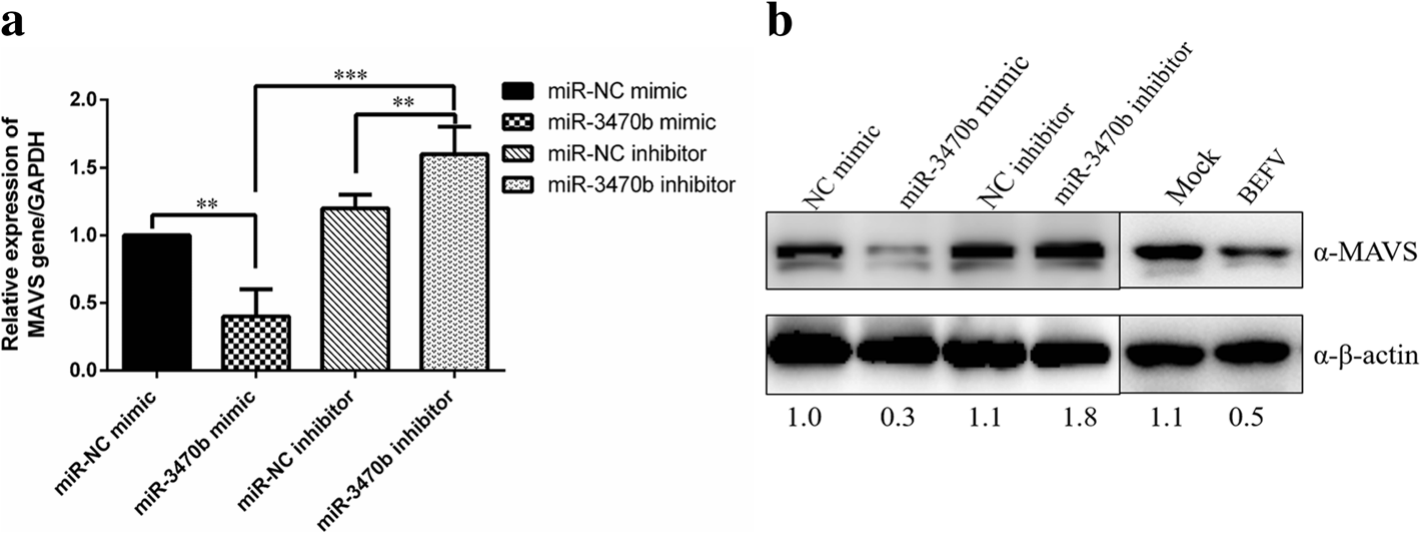
MiR-3470b downregulated the endogenous expression of MAVS. **a** RT-qPCR for detection of MAVS mRNA expression. The efficacy of the miR-3470b mimics or miR-3470b inhibitor and irrelevant-targeting negative control in regulating the mRNA levels of MAVS expression were detected by SYBR Green based RT-qPCR assay. **b** Western Blot for analysis of MAVS protein level. The cells transfected with miR-3470b mimics, miR-3470b inhibitor, irrelevant-targeting negative control and BHK-21 cells infected with BEFV (MOI of 0.1) for 36 h were harvested and subjected to analyze MAVS protein levels with rabbit anti-MAVS and β-actin antibodies for western blotting analysis. Numbers below the image was used for quantifications of protein blot intensities by gray value analysis derived from Image J software

To reveal the potential mechanism underlying the promotion of miR-3470b in BEFV replication, we next predicted the conceivable targets of miR-3470b using bioinformatics prediction software from databases such as Targetscan (http://www.targetscan.org), RNAhybrid (http://bibiserv.techfak.uni-bielefeld.de/rnahybrid/). Among those predicted targets, MAVS, the crucial role in the host innate immune response is well established [15]. We found that the 3’UTR of MAVS mRNA contained the putative miR-3470b seed-matching site, suggesting that MAVS may be a potential target for miR-3470b (Fig. 3a). Therefore, we hypothesized that miR-3470b-mediated promotion of BEFV replication may achieve through targeting MAVS. To verify the bioinformatics predication, the wild or mutant type containing six base pair mutations in the seed region of 3’UTR of MAVS was constructed and inserted into the pmirGLO dual-luciferase reporter vector (Fig. 3a). And then the luciferase reporter assay was conducted in 293 T cells co-transfected with this reporter plasmid and miR-3470b mimics or miR-NC mimics. As expected, decreased luciferase signal was observed following co-transfection of miR-3470b mimics and wild-type MAVS reporter plasmid, whereas no obviously inhibited luciferase activity was obtained following treatment with miR-NC mimics or 3’UTR of MAVS reporter plasmid with some mutated binding sites(Fig. 3b). To further investigate the influence of the interaction between miR-3470b and the MAVS 3’UTR on MAVS expression, RT-qPCR and western blot analysis were performed to measure endogenous MAVS expression upon miR-3470b mimics or inhibitors in BHK-21 cells. As predicted, over-expression of miR-3470b led to great decrease in MAVS both at the transcriptional and protein levels. On the contrary, miR-3470b inhibitors increased the expression of MAVS (Fig. 4a, b). Moreover, the direct effect of endogenous miR-3470b (not the mimic) on MAVS expression was investigated in BEFV-infected cells, it was also shown that BEFV infection suppressed MAVS expression (Fig. 4b).Therefore, these data indicate that MAVS is a direct target of miR-3470b, and its expression is partly inhibited by miR-3470b.

### MAVS expression in BHK-21 cells suppress the replication of BEFV

**Fig. 5:**
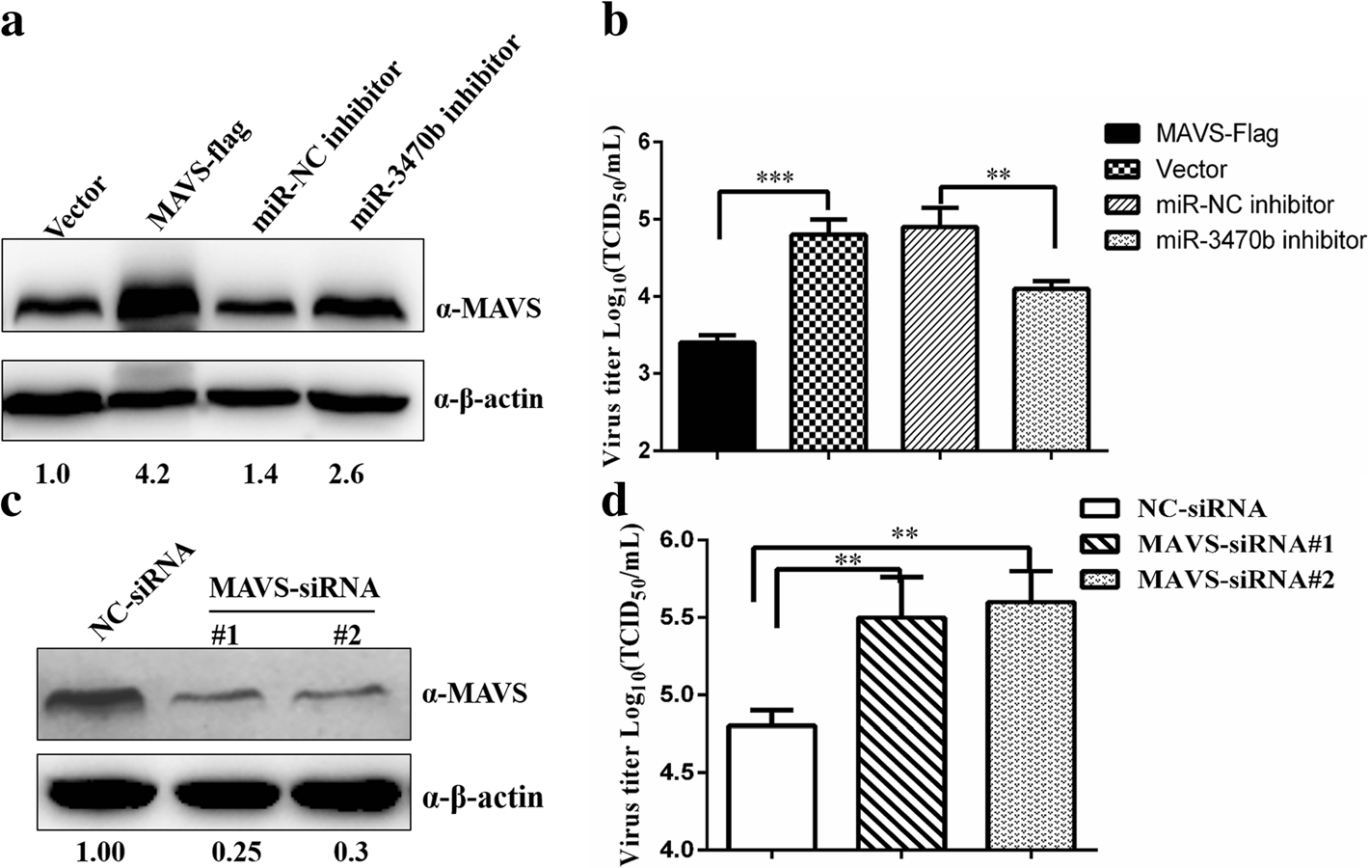
Expression of MAVS suppress BEFV replication in BHK-21 cells. **a** Detection of MAVS protein expression by western blot assays after transfection with flag-tagged MAVS recombinant plasmid, the miR-3470b inhibitor and corresponding control groups, respectively. **b** Flag-tagged MAVS recombinant vector, the miR-3470b inhibitor and corresponding control groups was transfected in BHK-21 cells followed by infection with BEFV at an MOI of 0.1 for 24 h. Then, viral titers in the cell cultures were measured by TCID_50_. Data are representative of three indcdent experiments and presented as means ± SD. ***p < 0.001, **p < 0.01, and *p < 0.05. **c** BHK-21 cells were transfected with siNC, siMAVS respectively, and then MAVS protein levels in siRNA-transfected cells were determined by western blotting. Numbers below the image was used for quantifications of protein blot intensities by gray value analysis derived from Image J software. **d** BHK-21 cells transfected with siNC, siMAVS#1 and siMAVS#2 for 24 h and then followed by infection with BEFV (0.1MOI). Subsequently, viral titers in the cell cultures were measured by TCID_50_. Data are representative of three independent experiments and presented as means ± SD. ***p < 0.001, **p < 0.01, and *p < 0.05

To test whether the expression of MAVS and miR-3470b expression had an opposite effect on BEFV replication. First of all, BHK-21 cells were transfected with flag-tagged MAVS recombinant plasmid, the miR-3470b inhibitor and corresponding control groups, respectively. The results of western blot assays showed that MAVS protein expression was significant up-regulated (Fig. 5a). Furthermore, the BHK-21 cells overexpressed MAVS or miR-3470b knock down with indicated miR-3470b inhibitor were infected with BEFV for 24 h. Contrary to the effect of miR-3470b on BEFV replication, the results of virus titration analysis indicated that cells in which MAVS overexpression had markedly reduced BEFV titers compared to the control cells, while a significant increasing trend in BEFV titers was observed in silenced miR-3470b cells compared to the control cells (Fig. 5b). Simultaneously, to further investigate the potential function of MAVS in BEFV biology. RNA interference was used to knockdown MAVS expression in BHK-21 cells, and the silence efficiency were determined by western blotting and the effect on BEFV replication was examined. The results showed that the siRNA knockdown of MAVS led to MAVS protein reduction when compared with the siRNA negative control (siNC) respectively (Fig. 5c). Furthermore, transfection with siMAVS also resulted in considerably increased BEFV titration (Fig. 5d). Taken together, the above data show that overexpression MAVS or silencing miR-3470b expression inhibits BEFV replication, whereas knockdown of MAVS exerted the opposite effects.

## Discussion

In recent years, high-throughput sequencing technology has been widely used to reveal the expression profiles of multiple miRNAs and mRNAs in virus-infected sample and analyzing those data comprehensively helps us to discover these functional molecules involved in complicated diseases [23, 24]. To further explore the replication mechanism of BEFV, we performed an integration analysis of miRNA and mRNA expression profiling of BEFV infected BHK-21 cells using a deep sequencing approach (data not shown), and tried to find out possible important regulatory mechanisms of BEFV replication mainly focusing on antiviral immunity.

MicroRNAs (miRNAs) are critical regulators of gene expression and accumulating evidences have revealed that cellular miRNA-mediated antiviral defense play an important role in the control of numerous virus infection and replication [25–27]. However, the role of miRNA in the process of BEFV infection has not been reported. In this study, firstly, we screened a miRNA pool covering 23 highly expressed miRNAs and further identified miR-3470b was significantly up-regulated highly expressed during virus infection using RT-qPCR (Fig. 1). To illustrate the effect of miR-3470b on MAVS expression and BEFV replication, the MAVS expression in miR-3470b mimics, inhibitors transfected or BEFV-infected cells was presented and miR-3470b expression indeed improved BEFV replication (Figs. 2&4), indicated that miR-3470b might have potential function in BEFV infection. Mounting evidence has shown that viruses can take advantage of host miRNAs to facilitate their survival and replication [27, 28]. Nevertheless, miR-3470b is a novel miRNA molecule, no literature is available to show its role in antiviral responses of host. Activation of the innate immunity provides the first and most important lines of defense against invading pathogens infection [29, 30]. So, given the importance of miRNA-3470b exerting its promoting virus replication function, we hypothesized that miRNA might also regulate type I IFN signaling to create a favorable environment for virus survival. Thus, we set up this experiment to verify these hypotheses.

It is reported that miRNAs post-transcriptionally regulate the expression of multiple genes by binding to the 3’UTR of their target messenger RNAs to play biological functions [31, 32]. Through bioinformatics software prediction of miR-3470b target, 3342 transcripts with sites were exhibited, which may be participate in numerous biological or pathological processes. Among those molecules, we discovered that MAVS might a direct target gene of miR-3470b and it was further confirmed by dual luciferase activity assay, because the presence of binding sites in target mRNAs does not guarantee actual miRNA binding due to mRNA secondary structure. (Fig. 3). So it is clear that miR-3470b at least, partly utilize sequence complementarity to bind and decrease the stability or translation efficiency of MAVS expression pattern. It is generally known that MAVS, as a key central hinge adaptor molecule, involved in antiviral or inflammatory responses or other signaling cascades [33]. Nevertheless, an accumulating body of evidence has shown that many intracellular pathogens employs multiple diverse mechanisms to regulate MAVS to counteract the host immunity, including protein–protein interactions for physical space-occupying of MAVS signaling partners [34], post-translational modifications such as phosphorylation and ubiquitination [18], inhibition or degradation of MAVS by negative regulator encoded by virus [35–37] and miRNA-mediated MAVS gene expression silencing [20, 21]. Thus, we conclude that the inhibitory of MAVS expression may be responsible for promoting BEFV effective replication.

To further elucidate the novel mechanism, we sought to restoration of MAVS expression by overexpression of MAVS, miR-3470b inhibitor or siRNA-MAVS during viral infection, and markedly suppressed the effect of on BEFV replication was observed, whereas knockdown of MAVS had the opposite effect (Fig. 5). The results indicate that enhance of MAVS expression in BEFV infection may potential provides a new clue for the effective prevention and control of BEFV. To the best of our knowledge, we identified a novel regulator of MAVS expression, miR-3470b inhibited MAVS by targeted its 3’UTR, ultimately contributed to BEFV replication. These findings have provided insights for further studies of the molecular mechanism underlying host response to BEFV infection. Nevertheless, the study on the function of miR-3470b and underlying regulatory mechanisms of MAVS during BEFV infection has not been reported as yet. In this study, we demonstrate that the miR-3470b enhances BEFV replication as evidenced by the significant difference in BEFV replication by either 3470b transfection or inhibition. But the effect of miR-3470b on levels of MAVS RNA and protein together with a similar reduction in the luciferase reporter experiment seems have the relatively small effect on MAVS protein (Figs. 2 and 4). So some inferences drawn from data indicating that MAVS is possible not the only BEFV-limiting target of miR-3470b. Furthermore, in view of the results obtained from direct effect of endogenous miR-3470b on MAVS expression in BEFV-infected cells (Fig. 4b), it was also shown that BEFV infection suppressed MAVS expression. Therefore, there is still much study needed to explore the in-depth mechanisms about miR-3470b function. What’s more, whether other factors have the similar effects with miR-3470b on MAVS expression is an interesting and important question deserving further research.

## Conclusions

In summary, our results revealed that BEFV infection could significantly up-regulate the miR-3470b expression. Prediction of miR-3470b targets led us to discover one of the miR-3470b response elements (MREs) in the 3’UTR of MAVS. Moreover, miR-3470b mediated suppression of MAVS expression contributed to BEFV replication. These findings not only provide new insights into virus and host interactions during BEFV infection, but also suggest potential new therapeutic targets and treatment strategies.

## Methods

### Cell lines, viral strain and animals

HEK-293T (GDC0067) obtained from China Center for Type Culture Collection (CCTCC); the Baby Hamster Syrian Kidney cells (BHK-21) lines provided by American Type Culture Collection (ATCC No.: CCL-10) were preserved in our laboratory. The BHK-21 and HEK-293 T cells were cultured in Dulbecco’s Modifed Eagle’s Medium (Gibco, Grand Island, NY, USA) supplemented with 10% fetal bovine serum (FBS), 100 U/ml penicillin, and 100 mg/ml streptomycin sulfate (Gibco, Grand Island, NY, USA) and maintained at 37 °C in an incubator containing 5% CO_2_. BEFV (Shandong/China/2011) were isolated and stored in the Ruminant Disease Research Center, Shandong Normal University, Jinan, Shandong Province, China. The 50% tissue culture infected dose (TCID_50_) of BEFV determined by Reed-Muench method have been described previously [38, 39]. Six adult male New Zealand white rabbits (mean body weight 2.0 kg) provided by the Experimental Animal Center Shandong Normal University were used for preparation of anti-BEFV-G1 polyclonal antibody.

### Production of anti-BEFV-G1 polyclonal antibody

At present, there is no commercialization of BEFV or viral protein antibodies that is available for study of BEFV. Reportedly, BEFV glycoprotein neutralization site 1 referred as G1 protein is a well-studied immunogenic region involving in eliciting robust antibodies, which can be used as a potential research tool for diagnostic reagents and novel antiviral agents [39]. In this study, anti-BEFV-G1 polyclonal antibody was prepared by immunizing pure New Zealand rabbits with purified His-fusion G1 proteins for analysis of BEFV G protein level in western blot. First, construction of recombinant plasmids and purification of BEFV recombinant G1 protein in *E. coli* have been described previously [39]. Then, 200 μg of recombinant His-G1 fusion protein mixed with an equal volume of Freund’s complete adjuvant (Sigma Aldrich, St. Louis, MO, USA) was first injected intradermally on the back and proximal limbs of rabbit. Ten days later, 200 μg of recombinant fusion protein in Freund’s incomplete adjuvant (Sigma Aldrich, St. Louis, MO, USA) were injected subcutaneously to boost immunization stimulation at 2 weeks interval, and the antiserum was collected from the carotid artery of the rabbits after the last strengthened injection for a week.

### Small RNA deep sequencing

BHK-21 cells was infected with BEFV at a multiplicity of infection (MOI) of 0.1 or mock infected for 24 h, and then the cells were harvested. Total cellular RNA was extracted using Trizol Reagent (Invitrogen, NY) according to the manufacturer’s protocol. The quality and quantity of total RNA were evaluated by using an Agilent 2100 Bioanalyzer (Agilent Technologies, USA) and Agilent RNA 6000 Nano kit, and then the RNAs were carried out small RNA deep sequencing (BGISEQ, BGI, Shenzhen). The data then were analyzed by BGI Co., Ltd.

### Quantification of miR-3470b expression

BHK-21 was infected with the BEFV (Shandong/China/2011) at a multiplicity of infection (MOI) of 0.1, meanwhile, BHK-21 was infected with different doses of BEFV ranging from 0.001 and 1 MOI, and total cell was collected at 24 h and 48 h post infection. Then, cell pellets with or without BEFV infection was subjected to miRNA extraction using a miRcute miRNA isolation kit (Tiangen Biotech Co., Ltd., Beijing, China). Reverse-transcribed to cDNA from 2 μg of total RNA was conducted according to the manufacturer’s instructions of the miRcute miRNA first-strand cDNA synthesis kit (Tiangen Biotech Co., Ltd., Beijing, China). MiR-3470b quantification was performed using the miRcute miRNA qPCR detection kit (SYBR Green) (Tiangen Biotech Co., Ltd., Beijing, China) on a Light Cycler 480 real time quantitative PCR system (Roche Applied Science) with a universal reverse primer and a specific forward primer. Real time PCR amplification was performed for 15 min at 95 °C, followed by 40 cycles of 94 °C for 20 s, 65 °C for 34 s, and dissociation at 95 °C for 15 s, 65 °C for 60 s, and 95 °C for 30 s with the following primer sequences:5’-GCCTCACTCTGTAGACCAGGCTGG-3′. U6 small nuclear RNA is a representative internal control for quantitating miRNA [28] with the primer sequences: 5’-GCTCGCTTCGGCAGCACATA-3′. Thus the relative expressions of mature miR-3470b was normalized to U6 within each sample and fold changes were calculated through relative quantification using the 2^-△△Ct^ method.

### Target gene prediction

The potential targets of miR-3470b (miRBase accession number MIMAT0015641 in the microRNA databases (http://www.mirbase.org/)) were predicted using different miRNA target gene prediction tools with TargetScan (http://www.targetscan.org/) and RNAhybrid (http:// bibiserv.techfak.uni-bielefeld.de/rnahybrid/).The potential relevance of predicted targets were taken the intersection.

### Construction of recombinant plasmid

The cDNA of *Mesocricetus auratus* was used as template for PCR amplifying the MAVS gene using sense primer MAVS-F and antisense primer MAVS-R. The PCR product was cloned into the sites of *EcoR* I and *Not* I of the pcDNA_3.1_(+) vector flanked by flag-tagged in the 5′ end of MAVS. Meanwhile, the MAVS 3’UTR sequence (MAVS 3’UTR WT) containing putative miR-3470b targeting seed match sites sequence (5′-CAGAGTG-3′) was amplified from genomic DNA by PCR, and the corresponding mutation sequence (5′-GTCTCAC-3′) (MAVS 3’UTR Mut) was created by overlap extension of PCR. A fragment consisting of wild-type 3’UTR of MAVS or site-directed mutagenesis 3’UTR of MAVS was inserted into of the dual-luciferase reporter vector pmirGLO (Promega Corporation, Madison, WI, USA) at the *Pme* I and *Xba* I restriction sites, respectively. The primers for amplification of the MAVS and MAVS 3’UTR gene were listed in Table 1.

**Table 1:** The sequences of primers used for construction of MAVS recombinant vector

Gene	Primer name	Sequences (5′-3′)	Size(bp)
MAVS	MAVS-*EcoR* I-F	CCGGAATTCGCCACCATG*GACTACAAGGACGACGATGACAAG*ACATTCGCTGAGGACAAG	1497
	MAVS-*Not* I-R	AAGGAAAAAAGCGGCCGCTCATTGGGCCAGGTGCCTACTACGGTAC	
MAVS 3’UTR WT	*Pme* I-F	AGCTTTGTTTAAACAGCCTCAGCTGCGTGCCGTTCGCT	657
	*Xba*I-R	CTAGTCTAGAGAGGTGGGGTTAGGAGGCTGGAGCA	
MAVS 3’UTR Mut	*Pme* I-F	AGCTTTGTTTAAACAGCCTCAGCTGCGTGCCGTTCGCT	481
	R1	GGAACCAGCGTGAGACCTGCTCCCAGGCTCTAGTA	
	F1	CAGGTCTCACGCTGGTTCCTAGGGAGCTTTG	177
	*Xba*I-R	CTAGTCTAGAGAGGTGGGGTTAGGAGGCTGGAGCA	

Note: Underlined and italic parts of primers reprensent restriction endonucleases sites and flag-tagged sequences, respectively

### Dual-luciferase reporter assay

For the reporter assays, 293 T cells were plated into 96-well plates and 50 nM miR-3470b mimic or 50 nM inhibitor or their respective non-targeting negative control oligonucleotides purchased from RiboBio (Guangzhou, China) was co-transfected with 0.5 μg of MAVS 3’UTR WT or MAVS 3’UTR Mut recombinant plasmids using the Attractene Transfection Reagent (Qiagen, Germany) for 36 h. The empty plasmid pmirGLO group was used for the negative control, and non-transfected 293 T cells were used as the blank control. Then Firefly and *Renilla* luciferase activities were measured using a dual-luciferase reporter assay system kit (Promega Corporation, Madison, WI, USA) using a Spectra Max M5 microplate reader (Molecular Devices Instruments Inc., USA) following the manufacturer’s instruction. Firefly luciferase activities was normalized against *Renilla* luciferase activities as the relative fluorescence intensity. All experiments were performed three times.

### RNA interference

To knockdown the expression of endogenous MAVS in BHK-21 cells, the small interfering RNAs (siRNAs) targeting MAVS were designed and synthesized by Biomics Biotechnologies Co., Ltd. The sequences of siRNAs with dTdT overhangs were as follows, sense: 5′-CAGUGACCAGGAUCGACUAdTdT-3′ and antisense: 5′- UAGUCGAUCCUGGUCACUGdTdT-3′ for siMAVS#1, sense:5′- GGUGACACCUCCACAGCAAdTdT-3′ and antisense: 5′- UUGCUGUGGAGGUGUCACCdTdT-3′ for siMAVS#2. Negative siRNA with scrambled sequences sense: 5′-UUCUCCGAACGUGUCACGUdTdT-3′ and antisense: 5′-ACGUGACACGUUCGGAGAAdTdT-3′ were used as a negative control (siNC). siRNAs were transfected into BHK-21 cells at 50 nM by using Attractene Transfection Reagent (Qiagen, Germany) according to the manufacturer’s instructions. At 48 h post-transfection, cells were harvested and lysed and immuoblotted with anti-rabbit MAVS Ab (CST, Proteintech Group, Chicago, IL, USA) to determine the knockdown efficiency of MAVS.

### RT-qPCR for monitoring MAVS RNA levels

BHK-21 cells were plated and cultured to a 70% confluence in DMEM before 50 nM miR-3470b mimic, 50 nM of the miR-3470b inhibitor, 50 nM irrelevant-targeting negative control mimic (NC) (Ribobio, Guangzhou, China) were transfected for 36 h. The cells were harvested, and total RNA extraction and first-strand cDNA synthesis was performed as previously described [39]. The mRNA levels of MAVS were detected by SYBR Green based RT-qPCR assay using a Light Cycler 480 (Roche Applied Science, USA) according to the manufacturer’s protocol. The sequences of the primers used for MAVS quantitation were as follows: F: 5’-TTTGCTGTCTTGACGTTTTGGA-3′, R: 5’-CGGTTCCCTAGTTGTGTGTAGGA-3′. A house keeping gene glyceraldehyde-3-phosphate dehydrogenase (GAPDH) is commonly used as an internal control for quantitating mRNA [39]. Therefore, for monitoring MAVS RNA levels of all samples, GAPDH gene was used to normalize the endogenous input RNA and fold changes were analyzed through relative quantification by the 2^-ΔΔCt^ method [40].

### Western blotting analysis of MAVS expression

BHK-21 cells (1 × 10^5^ cells per well) were seeded into six well culture plates, incubated overnight and transfected with 50 nM of the miR-3470b mimics or 50 nM of the miR-3470b inhibitor or their respective non-targeting negative control oligonucleotides using Attractene Transfection Reagent (Qiagen, Germany). Meanwhile, BHK-21 cells was infected with BEFV at a multiplicity of infection (MOI) of 0.1 were harvested at 36 h post-transfection and subjected to analyze MAVS protein levels with rabbit anti-MAVS (CST, Proteintech Group, Chicago, IL, USA) and β-actin (Abways, China) antibodies for western blotting analysis. The blots were detected by ECL reagent (Thermo Scientific, USA) and scanned by the enhanced chemiluminesence detection system (Amersham, USA). Image J software was used for quantifications of protein blot intensities by gray value analysis (National Institutes of Health, Bethesda, MD, USA).

### BEFV replication levels analysis

BHK-21 cells were inoculated with 0.01MOI BEFV after transfection with 50 nM of miR-3470b mimic, NC mimic, miR-3470b inhibitor and NC inhibitor respectively at the indicated time points. After three times freeze-thaw, cells and culture medium were harvested for virus titration expressed as the lgTCID_50_/ml by the Reed-Muench endpoint method. Meanwhile, BEFV N gene mRNA levels were determined by real-time RT-PCR assay [39]. All samples were run in triplicate and fold changes were calculated using the 2^-ΔΔCt^ method. What’s more, BEFV G protein levels were analyzed by western blot as described above using the prepared rabbit anti-G1 polyclonal antibodies with 1/128 virus neutralization titer. Briefly, BEFV infected cells were lysed, and total proteins were subjected to western blot probed with rabbit anti-G1 polyclonal antibodies at a 1: 500 dilution or an anti-β-actin antibody at a 1: 5000 dilution (Abcam, USA). Then, HRP-conjugated anti-rabbit IgG (Jackson, USA) at a 1:3000 dilution was used to label primary antibody. Immunolabelled proteins were visualized using ECL reagent (Pierce).

### Statistical analyses

Statistical analyses were performed using the GraphPad Prism software (version 5.0; San Diego, CA, USA). Statistical significance was calculated by two-way ANOVA multiple comparisons test were made in grouped graphs and one-way ANOVA multiple comparisons test were made across multiple samples. The results are presented as the mean values ± standard deviation (SD) from at least three independent experiments, and *P* value below 0.05 was considered statistically significant (*, *P* < 0.05; **, *P* < 0.01; ***, *P* < 0.001).

## Additional file

Additional file 1: **Figure S1.** Statistic of differently expressed miRNAs (TIF 110 kb)

## Abbreviations

BEF: Bovine ephemeral fever
BEFV: Bovine ephemeral fever virus
BHK-21: Baby Hamster Syrian Kidney
Cardif: CARD adaptor inducing IFN-β
DMEM: Dulbecco’s modified Eagle’s medium
FBS: Fetal bovine serum
GAPDH: Glyceraldehyde 3-phosphate dehydrogenase
IAV: Influenza A virus
IPS-1: IFN-β promoter stimulator 1
JEV: Japanese encephalitis virus
MAVS: Mitochondrial antiviral signaling protein
miRNAs: MicroRNAs
MOI: Multiplicity of infection
NC: Negative control mimic
PBS: Phosphate buffered saline
RIG-I: Retinoic acid-inducible gene I
RT-PCR: Reverse transcription polymerase chain reaction
TCID_50_: 50% Tissue culture infected dose
VISA: Virus-induced signaling adaptor
