# Supplementary figures and images for: MiR-3470b promotes bovine ephemeral fever virus replication via directly targeting mitochondrial antiviral signaling protein (MAVS) in baby hamster Syrian kidney cells
Source: BMC Microbiol. 2018 Dec 27;18:224. doi: 10.1186/s12866-018-1366-6 (PMC6307158; doi:10.1186/s12866-018-1366-6)

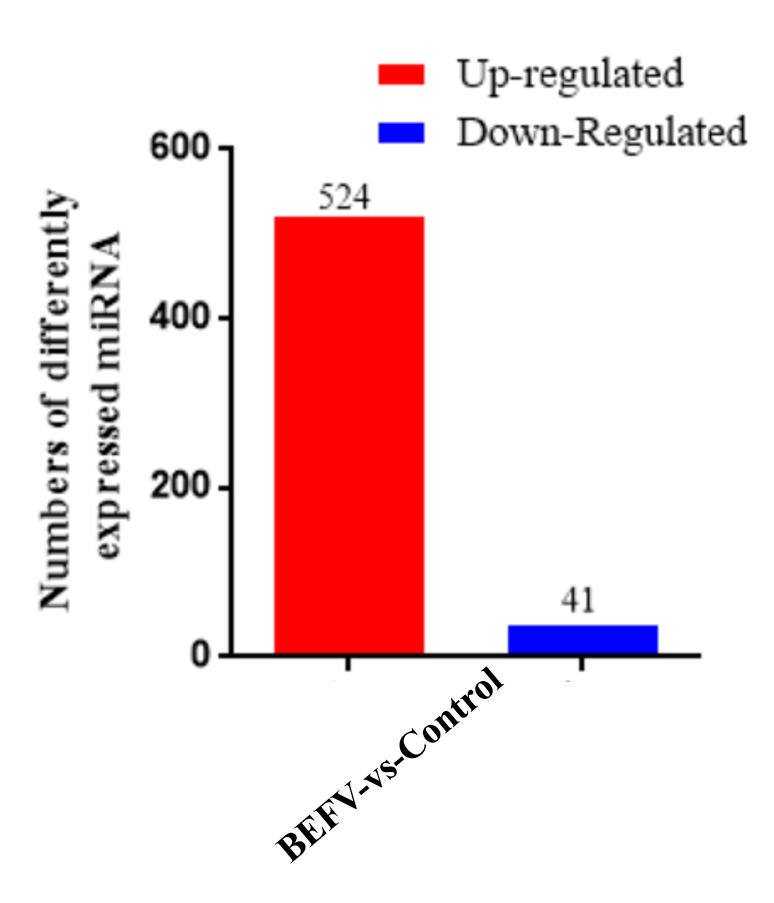

Supplement: Supplementary file 1 — Figure S1. Statistic of differently expressed miRNAs (TIF 110 kb) [file 12866_2018_1366_MOESM1_ESM.tif]
